# Molecular Docking and Kinetic Studies of the A226N Mutant of *Deinococcus geothermalis* Amylosucrase with Enhanced Transglucosylation Activity

**DOI:** 10.4014/jmb.2003.03066

**Published:** 2020-05-26

**Authors:** Seungpyo Hong, Inonge Noni Siziya, Myung-Ji Seo, Cheon-Seok Park, Dong-Ho Seo

**Affiliations:** 1 Research Group of Healthcare, Korea Food Research Institute, Wanju 55365, Republic of Korea; 2 Department of Food Science and Technology, College of Agriculture and Life Sciences, Jeonbuk National University, Jeonju 54896, Republic of Korea; 3 Division of Bioengineering, Incheon National University, Incheon 22012, Republic of Korea; 4 Graduate School of Biotechnology and Institute of Life Science and Resources, Kyung Hee University, Yongin 17104, Republic of Korea; 5 Department of Agricultural Convergence Technology, Jeonbuk National University, Jeonju 54896, Republic of Korea

**Keywords:** Transglucosylation, amylosucrase, *Deinococcus geothermalis*, 4-methylumbelliferone, molecular dynamics, docking simulation

## Abstract

Amylosucrase (ASase, E.C. 2.4.1.4) is capable of efficient glucose transfer from sucrose, acting as the sole donor molecule, to various functional acceptor compounds, such as polyphenols and flavonoids. An ASase variant from *Deinococcus geothermalis*, in which the 226^th^ alanine is replaced with asparagine (DgAS-A226N), shows increased polymerization activity due to changes in the flexibility of the loop near the active site. In this study, we further investigated how the mutation modulates the enzymatic activity of DgAS using molecular dynamics and docking simulations to evaluate interactions between the enzyme and phenolic compounds. The computational analysis revealed that the A226N mutation could induce and stabilize structural changes near the substrate- binding site to increase glucose transfer efficiency to phenolic compounds. Kinetic parameters of DgAS-A226N and WT DgAS were determined with sucrose and 4-methylumbelliferone (MU) as donor and acceptor molecules, respectively. The *k*_cat_/*K*_m_ value of DgAS-A226N with MU (6.352 mM^-1^min^-1^) was significantly higher than that of DgAS (5.296 mM^-1^min^-1^). The enzymatic activity was tested with a small phenolic compound, hydroquinone, and there was a 1.4-fold increase in α-arbutin production. From the results of the study, it was concluded that DgAS-A226N has improved acceptor specificity toward small phenolic compounds by way of stabilizing the active conformation of these compounds.

## Introduction

Glycosylation is widely used to improve the water solubility and oxidation stability of functional compounds [1- 3]. Transglucosidases are conventionally used for transglycosylation reactions but they require expensive donor molecules, such as UDP-glucose [4]. Amylosucrase (ASase, E.C. 2.4.1.4.) is a glycoconjugated enzyme belonging to the glycoside hydrolase 13 family that has the distinctive ability to synthesize α-1,4-glucans using sucrose as its sole substrate [5]. The enzyme has great industrial potential because it utilizes sucrose, a relatively cheap and economical donor molecule, for transglycosylation in highly efficient reactions [6-8]. One limitation of ASase is its limited selectivity for acceptor molecules, but it was demonstrated that the enzyme can transfer glucose to various hydroxylated molecules, including various monosaccharide derivatives such as salicin, arbutin, catechin, and hydroquinone (HQ) [6-10]. In addition, ASase can be used to increase the water-solubility and absorption rate of flavones, as was observed with baicalein, through the transfer of a glucoside to produce baicalein-6-O-α-glucoside (α-BG) [11].

ASase from *Deinococcus geothermalis* (DgAS) is reported to be able to catalyze transglucosylation reactions of various acceptor compounds [5]. The ASase molecule is composed of five domains (N, A, B, B’, and C) and it is domain A that provides a unique active site pocket where a sucrose molecule binds and glycosylation of acceptor compounds takes place [12]. Domain B forms a part of the pocket that can interact with substrate molecules. The domain in DgAS was found to be longer when compared to ASase from *Neisseria polysaccharea* (NpAS), with DgAS being shown to possess a wider active pocket [13]. A DgAS variant in which the B domain was substituted with the B domain of NpAS, showed increased transglucosylation activity compared to the wild-type (WT) DgAS, due to changes in the flexibility of loop structures [14]. Recently, a DgAS variant (DgAS-A226N) was constructed by substituting an arginine for the 226th amino acid, alanine, of the DgAS. Its substrate affinity to sucrose was increased by 2.0-fold and its polymerization activity was increased compared to that of WT DgAS [15]. While the polymerization capability of the mutation was proved in that study, this research investigated how the mutation increased the catalytic activity of the enzyme with particular focus on modulation of the enzyme activity. First, the interaction between DgAS-A226N and several acceptor compounds was evaluated by molecular dynamics (MD) and molecular docking simulations. Next, the kinetic parameters of acceptor and donor molecules in the transglycosylation reaction were measured for sucrose and 4-methylumbelliferone (MU) as donor and acceptor molecules, respectively. Finally, the production yields of the transglycosylation reactions associated with DgAS- A226N and WT DgAS were compared.

## Materials and Methods

### Structure Modeling

The crystal structure of DgAS in complex with turanose (PDB ID 3UER) was retrieved from the PDB databank [13]. The protein part of the structure was used for the simulation and for modeling the structure of DgAS-A226N. In order to model the covalently bound glucose moiety, the coordinates of the glucose moiety in turanose and that of aspartate at position 284 were extracted from the structure. These coordinates were loaded in Avogadro (version 1.2.0) [16], a covalent bond was generated between the glucose moiety and the aspartate, and the geometry of the resultant structure was optimized. The force field parameters of the glucose-aspartate conjugate were created using the AmberTools19 package [17]. For structure modeling of the mutant, the DgAS-A226N variation was introduced into the protein structure by using the fixed backbone application (*fixbb*) of the Rosetta suite (version 3.9) [18]. The structure and topology of the MD simulation were prepared with the Amber03 force field and the TIP3P water model was utilized to generate the solvated structure. This was followed by charge neutralization of the system through the addition of counter ions, a procedure executed using the *tleap* module available in the Amber suite [17].

### Molecular Dynamics (MD) Simulation

MD simulations were performed in Gromacs suite (version GROMACS 2020) [19]. The system was stabilized by the steepest descent method of energy minimization, and 100 ps each of NVT and NPT equilibration simulations were completed with position constraints for the heavy atoms of the protein molecule. The main simulation of 5 ns was implemented under the LINCS constraint and a V-rescale thermostat set at 300 K. The parameters of the simulation are listed in Table S1. Five independent simulations were executed for each of the WT DgAS and DgAS-A226N systems.

### Ensemble Docking Simulation

The structure ensemble was constructed by retrieving the conformations from the MD simulation trajectory with 50 ps intervals, and the ensemble consisted of 505 conformations. The conformations were converted into PDBQT format files using the AutoDock script *prepare_receptor4.py* [20]. The structures of the acceptor ligands were downloaded from PubChem with the following PubChem CIDs: 5280567 (4-methylumbelliferone, MU), 785 (HQ), 5280863 (kaempferol), 5281708 (daidzein), and 5280961 (genistein). These structures were converted to PDBQT format using the AutoDock script *prepare_ligand4.py*. Docking simulations were executed for each ligand and for each conformation of the ensemble using AutoDock Vina (version 1.1.2) [21]. The coordinates near the glucose moiety were used as the center of the docking simulations and the docking was executed in an isotropic box with a length of 15 Å, and up to 20 docking conformations were sampled.

### Software

Plots were generated using the Python Matplotlib package (version 3.1.1) [22]. The molecules were rendered in PyMol (version 2.2.2) (Schrödinger, LLC; http://www.pymol.org). A *t*-test was executed using the Python SciPy package (version 1.3.2).

### Kinetic Analysis of Action of DgAS-A226N and WT DgAS on MU

Purified WT DgAS and DgAS-A226N were obtained as described in previous studies [15]. Enzymatic assays were carried out in a total volume of 200 μl of 50 mM Tris-HCl (pH 8.0) containing 15 μg of the purified enzyme. The kinetic parameters *k*_cat_ and *K*_m_ were determined using both sucrose and MU as variable substrates. For the determination of *K*_m_ for MU, the concentration of sucrose was kept constant at 200 mM and that of MU varied from 0.1 to 1.0 mM. In contrast, for the determination of *K*_m_ for sucrose, the concentration of MU was kept constant at 1 mM and that of sucrose was varied from 0.4 to 200 mM. Each experiment was performed in triplicate. Initial velocities were fitted to the Lineweaver–Burk plot of Michaelis–Menten kinetics using Sigma Plot 13 (Systat Software, Inc., USA). Saturation was not achieved with some of the enzymes used and in those cases, the parameter *k*_cat_/*K*_m_ was calculated by linear regression analysis of the velocity versus substrate concentration plot (Figs. S3 and S4). After the enzyme reaction, the concentration of the product was determined by high- performance liquid chromatography (HPLC) analysis.

### High-Performance Liquid Chromatography (HPLC) Analysis

Detection and identification of bioconversion products such as 4-methylumbelliferone-7-*O*-α-D-glucopyranoside (MUG) and α-arbutin in the reaction mixtures were achieved by HPLC analysis. HPLC analysis was performed with a Zorbax Eclipse XDB-C18 column (5 μm, 4.6 × 250 mm; Agilent, USA) connected to a Shimadzu LC10AD_vp_ system (Shimadzu, Japan), a SPD-10A UV-VIS detector (set at 320 and 280 nm for 4-methylumbelliferone-7-*O*-α- MUG and α-arbutin, respectively), and an SPD-LC10 pump. Separation of reaction products was achieved with a gradient of 10-90% acetonitrile in 0.1% formic acid/water for 30 min at a flow rate of 1.0 ml/min. All solvents were filtered, degassed, and stored under pressure.

## Results and Discussion

### MD and Docking Simulations of WT DgAS and DgAS-A226N

**Fig. 1:**
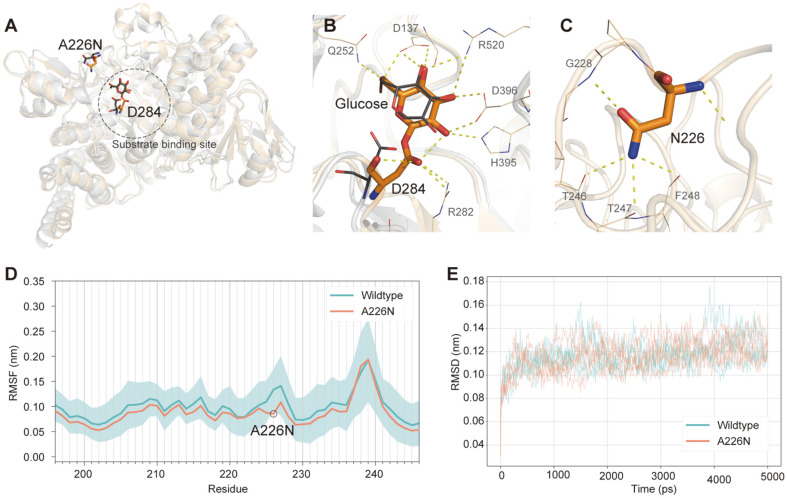
Structural models of DgAS and molecular dynamics (MD) simulation. Structural models of the WT DgAS and DgAS-A226N variants were created for the purpose of MD simulations. (**A**) The last snapshot of the simulation (pale orange) was almost identical to that of the initial structure (gray). D284 is located at the center of the substrate-binding site, whereas the A226N mutation is located on a loop near the enzyme active site that covers the substrate-binding site. (**B**) The glucose moiety was modeled to covalently bind to D284. Nonetheless, the conformation of the glucose moiety was retained after the simulation. The residue is shown in narrow gray and wider orange sticks for the initial and final snapshots of the MD simulation. (**C**) The asparagine on the 226^th^ residue was observed to form hydrogen bonds with nearby main chain atoms. (**D**) RMSFs were calculated for five independent molecular dynamics simulations, and the average values for the WT DgAS and mutant structures were computed. The 95% confidence interval (*t*-test) of the average RMSF is displayed as the shaded area. (**E**) RMSDs of the MD simulations.

Using computational approaches, we investigated how the A226N mutation affected the structure of the enzyme and changed its interaction with the ligand. In NpAS, the aspartic acid on residue 286 forms a covalent bond with the glucose moiety of sucrose and transfers it to the acceptor substrate [12]. The residue (Asp284 in DgAS) is located at the center of the substrate-binding site and the glucose moiety forms multiple hydrogen bonds with the enzyme (Figs. 1A and 1B). The DgAS-A226N mutation is located at the part of the loop that partially covers the substrate-binding site and is placed near the residue 284. The structural models for the WT DgAS and DgAS-A226N were created based on the crystal structure of the WT variant co-crystallized with turanose [13]. The overall structure of the enzyme remained stable after the MD simulation (Figs. 1A and 1E) and the localization of the glucose moiety was also unchanged after the simulation (Fig. 1B). The last snapshot of the MD simulation showed that the asparagine on residue 226 formed hydrogen bonds with atoms from the main chain of nearby loops (Fig. 1C). The root mean square fluctuation (RMSF) of the residue was significantly reduced by the mutation, and those of neighboring residues were also decreased (Fig. 1D). The flexibility of the other residues was also reduced in the mutant models, though the flexibility reduction was not statistically significant in most of these residues (Fig. S1). These results indicate that the mutation decreased the flexibility of the loop region located near the active site, which could affect the interaction with substrates. In a previous study, an MD simulation of the sucrose-enzyme interaction showed that the design of the active site promotes the formation of glycosidic links. This results in the lodging of the glucose moiety inside the active site with the fructose unit being excluded and then detached [23]. In the present study, the enzyme–acceptor substrate interaction was inspected for both WT DgAS and DgAS-A226N by employing an ensemble docking approach. The structure ensemble was retrieved from the MDS trajectories, and five phenolic substrates; MU, daidzein, genistein, HQ, and kaempferol, were docked to the ensemble. Glucose transfer would take place when the hydroxyl group of the substrate is situated between the carbon atom of glucose moiety and the glutamate residue 326 [12].

**Fig. 2:**
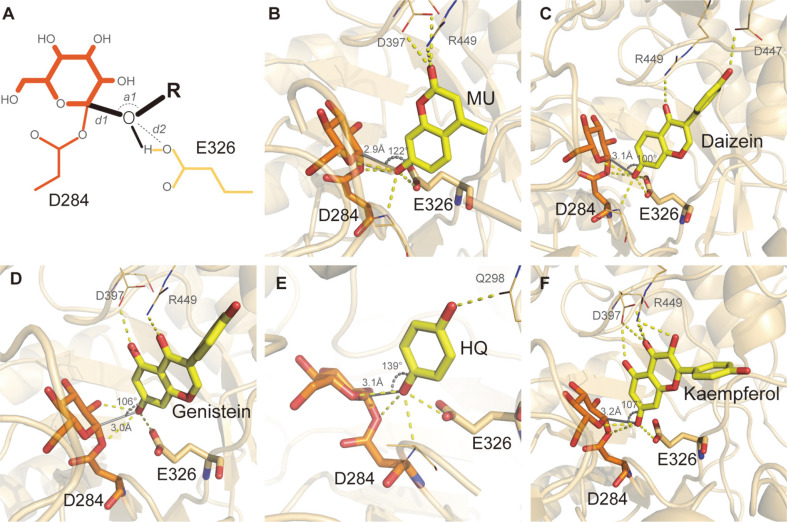
Docking conformations of phenolic substrates. Five phenolic substrates were docked to the ensemble structures of the WT DgAS and DgAS-A226N. (**A**) The glucose transfer reaction requires placement of atoms in specific coordinates. The hydroxyl group of the substrate should be placed close to the carbon atom of the glucose moiety that links the latter to the aspartic acid. Thus, the distance between this carbon and the hydroxyl group of oxygen of the substrate (*d1*) should be short enough to enable the reaction. Additionally, the angle (*a1*) between the hydroxyl group and the glucose moiety should be within a certain range. Moreover, the carboxylic group of E326 should be positioned close to the hydroxyl group of the substrate, which could be evaluated by the distance (*d2*) between the hydroxyl oxygen atom and any oxygen atom of the carboxylic group. Docking conformations that satisfied the conditions *d1* < 4Å, *d2* < 4Å, and 80° < *a1* <140° have been represented in panels B (MU), C (daidzein), D (genistein), E (HQ), and F (kaempferol).

**Fig. 3:**
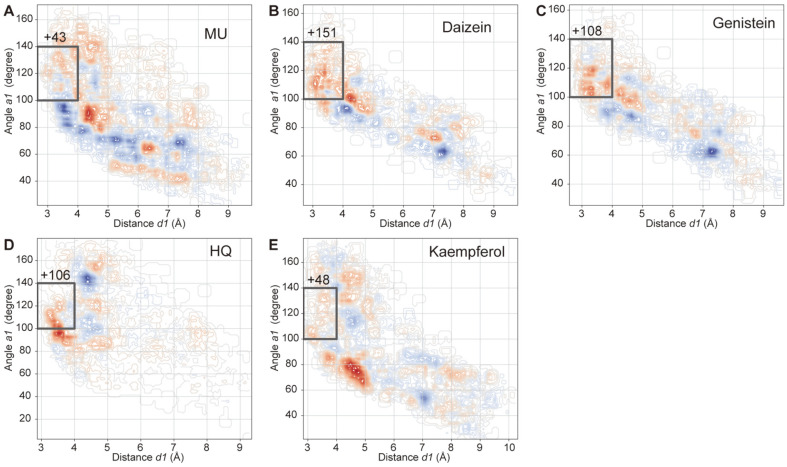
Differential conformation space between WT DgAS and DgAS-A226N. The docked conformations were mapped onto the two-dimensional space with the distance *d1* and angle *a1*, and the differences of conformational distributions for mutant and WT DgAS were plotted as contours, where mutant-enriched and -depleted regions were colored in red and blue, respectively. The docking results for (**A**) MU, (**B**) daidzein, (**C**) genistein, (**D**) HQ, and (**E**) kaempferol have been shown. Only conformations with *d2* smaller than 4 Å were analyzed. The region corresponding to the active conformation was marked by a gray box and the increased number of conformations in this region was displayed on top of the box.

Three geometric features, two distances (*d1* and *d2*) and one angle (*a1*), were measured to evaluate the possibility of glucose transfer (Fig. 2A). Docking poses that satisfied the criteria, including *d1* < 4 Å, *d2* < 4 Å, and 80o < *a1* <ere considered as reactive conformations that would enable the glucose transfer reaction. In all five substrates, conformations satisfying these conditions were found, and some of these have been illustrated in Figs. 2B-2F. The number of reactive conformations was also quantified. Two-dimensional plots of the docking conformations with *d1* and *a1* axes showed that the overall distribution of docking conformations was similar between WT DgAS and DgAS-A226N (Fig. S2). However, the DgAS-A226N variant was associated with altered distribution of the docking conformations, and the population of reactive conformations was higher in the mutant than in the WT DgAS (Fig. 3). These changes were consistently observed in all five phenolic compounds, suggesting that the mutation induces conformational changes in the enzyme, which favors enzyme interaction with phenolic compounds.

### Kinetic Parameters of WT DgAS and DgAS-A226N in the Presence of MU Acceptors

The kinetic parameters of the enzymatic reaction of WT DgAS and DgAS-A226N with sucrose and MU as donor and acceptor, respectively, were examined at 45°C. Although *K*_m_ values for DgAS in the presence of MU did not display saturation kinetics, those for DgAS-A226N under the same conditions could be extracted using the Michaelis-Menten equation. Both enzymes were saturated with a range of sucrose concentration and a fixed MU concentration. The steady-state kinetic constants determined this way are summarized in Table 1. The *K*_m_ values for WT DgAS and DgAS-A226N in the presence of sucrose were 36.17 and 44.57 mM, respectively. Although the *K*_m_ value for DgAS-A226N associated with sucrose was higher than that for WT DgAS, the difference was not significant. Furthermore, the *V*_max_ value for DgAS-A226N in the presence of sucrose (0.093 μmole/min/mg) was similar to that of WT DgAS (0.090 μmole/min/mg). These results indicate that the *k*_cat_ values of WT DgAS and DgAS-A226N were similar, suggesting that the donor binding affinity and catalytic efficiency of these variants were identical. The overall performance was expressed as *k*_cat_/*K*_m_ and that of WT DgAS toward MU was determined to be 5.296 mM-1min-1 by linear regression. The *k*_cat_/*K*_m_ value for DgAS-A226N under similar conditions was found to be higher (6.352 mM-1min-1) than that of WT DgAS. The *K*_m_ and *k*_cat_ values of DgAS- A226N reacting with MU were 2.718 mM and 17.267 min-1, respectively. The implications of these findings were compared to those of a previous study in which OleD, an oleandomycin glycosyltransferase from *Streptomyces antibioticus*, was not saturated with the MU acceptor, while the OleD triple mutant was saturated [24]. It was found that the OleD triple mutant exhibited not only increased specific activity with MU as an acceptor and UDP- glucose as a donor, but also possessed an improved glycosylation rate for various phenolic compounds, including kaempferol, daidzein, and genistein [24]. In the case of the A226N mutation however, the specific activity for MUG production of DgAS-A226N (0.067 ± 0.004 μmole/min/mg) was comparable to that of WT DgAS (0.063 ± 0.002 μmole/min/mg), with 200 mM sucrose and 1 mM MU as donor and acceptor, respectively. These results indicated that, although the specific activity for transglycosylation of DgAS-A226N did not increase relative to that of the WT, the acceptor specificity of DgAS-A226N might have improved due to the mutation.

In another study, Kim *et al*. constructed a β-glycosidase variant (2F6) with increased glycosynthase activity and carried out a kinetic investigation using *p*-nitrophenyl-β-D-glucopyranoside, *p*-nitrophenyl-β-D-mannopyranoside, and *p*-nitrophenyl-β-D-xylopyranoside as acceptors [25]. The kinetic parameters of *p*-nitrophenyl-β-D-xylopyranoside could not be estimated, but the *K*_m_ value for 2F6 with *p*-nitrophenyl-β-D-xylopyranoside as an acceptor showed a significant increase [25], indicating that the acceptor binding affinity of 2F6 for *p*-nitrophenyl- β-D-xylopyranoside was lower than that of the wild-type enzyme. Likewise, in this study, the A226N variant showed an increase in the Km value which suggests lower affinity for the substrate but with the corresponding results, also implies that the mutant developed an inclination to more specific substrates in regard to their form and structure. In addition, while the mutant *V*_max_ is slightly higher, it was not significantly different from the WT DgAS. The high affinity of the WT DgAS means that the enzyme binds more strongly to the substrate but may also require more energy to release it after catalysis, which could lead to lower production.

### Bioconversion of Hydroquinone (HQ) to α-Arbutin by WT DgAS and DgAS-A226N

**Fig. 4:**
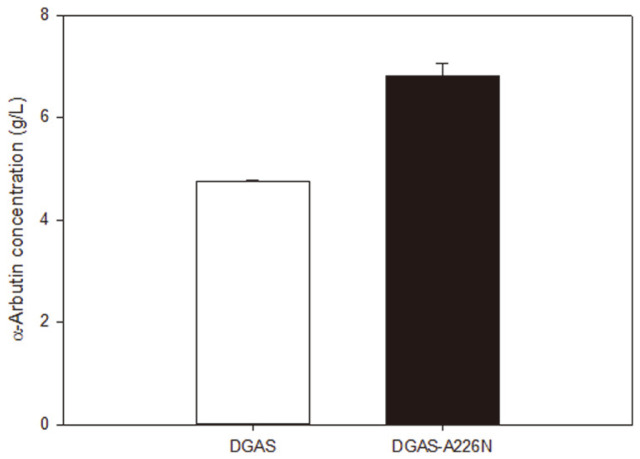
Production rate of α-arbutin by WT DgAS and DgAS-A26N with hydroquinone (HQ) as an acceptor and sucrose as a donor.

The kinetic parameters of MU were studied and it was revealed that while specific activity did not increase when compared to the wild type, the mutant had the possibility of improving acceptor specificity. To test this theory, a small phenolic compound was used in the form of hydroquinone and taken as an acceptor molecule. The assumed selectivity was based on the docking poses which were run on five phenolic substrates and indicated that the glucose molecule would be transferred in the case of the hydroxyl group of a substrate occurring between the carbon atom of glucose moiety and the glutamate residue 326. As a previous study had already generated α-arbutin through bioconversion using WT DgAS acting on hydroquinone, it was the ideal compound to investigate the bioconversion capabilities caused by the mutation. The lowered enzyme flexibility toward the active site supports the selectivity of acting on specific compounds, implying that phenolic compounds, bearing similar conformations, will succumb to transglycosylation and polymerization under the DgAS-A226N mutant predilection. Thus, in order to confirm that the acceptor specificity of DgAS-A226N had improved, hydroquinone (HQ) was used. Seo *et al*. had earlier generated α-arbutin using WT DgAS with HQ as an acceptor, sucrose as a donor, and ascorbic acid as an antioxidant to prevent the oxidation of HQ [26]. Although the conversion yield of hydroquinone to α-arbutin by WT DgAS was over 90%, a high molar ratio of donor to acceptor was required. We hypothesized that DgAS-A226N might produce more α-arbutin than WT DgAS under the same reaction conditions, if its acceptor specificity had improved. WT DgAS and DgAS-A226N reactions were carried out with sucrose, HQ, and ascorbic acid in a molar ratio of 1:1:0.1 (100 mM:100 mM:10 mM). Afterwards, the quantity of α-arbutin was analyzed by HPLC (Fig. 4). The production rate of α-arbutin by DgAS-A226N was increased by 1.4-fold (6.80 ± 0.26 g/l) in comparison to that of WT DgAS (4.76 ± 0.02 g/l). The aforementioned OleD variant, whose acceptor specificity was improved, displayed 2.1-, 5.7-, and 7.7-fold increased glycosylation rates toward kaempferol, daidzein, and genistein, respectively, in comparison to those of the wild-type enzyme [24]. In the case of DgAS, not only was DgAS-A226N identified to be the most thermostable ASase, but its acceptor specificity was also improved toward small phenolic compounds. This suggests that the WT DgAS would not be particularly stable. In agreement with this observation, computational approaches showed that the mutation would reduce the flexibility of the enzyme, especially near the active site.

Enzyme kinetics showed that the mutation facilitated the interaction with the acceptor molecule while the ligand docking simulations indicated that the mutation would increase the population of conformations that enable glucose transfer reactions for all phenolic compounds. Altogether, the introduced asparagine residue would interact with the main chain and reduce the flexibility of the enzyme in its vicinity, and that change would be expected to shift the conformational population of the enzyme in a manner in which its interaction with phenolic compounds is favored.

## Supplemental Materials



Supplementary data for this paper are available on-line only at http://jmb.or.kr.

## Figures and Tables

**Table 1:** Kinetic parameters of WT DgAS and DgAS-A226N in the presence of sucrose and MU at 45°C.

DgAS	Acceptor (MU)	Donor (sucrose)
*K_m_* (mM)	-	36.174 ± 2.037
*V_max_* (μmol/min•mg)	-	0.090 ± 0.002
*k_cat_* (min-1)	-	6.574
*k_cat_*/*K_m_* (min-1•mM)	5.296	0.182
Behavior	Linear	Saturation
DgAS-A226N	Acceptor (MU)	Donor (sucrose)
*K_m_* (mM)	2.718 ± 0.655	44.570 ± 4.625
*V_max_* (μmol/min•mg)	0.236 ± 0.044	0.093 ± 0.004
*k_cat_* (min-1)	17.267	6.836
*k_cat_*/*K_m_* (min-1•mM)	6.352	0.153
Behavior	Saturation	Saturation

-: Not detected
