# Supplementary material for: Molecular Docking and Kinetic Studies of the A226N Mutant of Deinococcus geothermalis Amylosucrase with Enhanced Transglucosylation Activity
Source: J Microbiol Biotechnol. 2020 May 26;30(9):1436–42. doi: 10.4014/jmb.2003.03066 (PMC9728394; doi:10.4014/jmb.2003.03066)
Supplement: Supplementary file 1 [file JMB-30-9-1436-supple.pdf]

J. Microbiol. Biotechnol.
https://doi.org/10.4014/jmb.2003.03066

Table S1. Molecular dynamics simulation parameters

Parameter	Value
Simulation suite	GROMACS 2020
Force field	AMBER03
Water model	TIP3P
Coulomb type	PME
VDW type	Cut-off
VDW cutoff	1 nm
Reference temperature	300 K
Temperature coupling	V-rescale
Pressure coupling	Parrinello-Rahman
Pressure coupling type	Isotropic
Constraints	All-bonds
Constraint algorithm	LINCS
Time step	2 fs
Number of steps	2,500,000
Structure sampling interval	5000

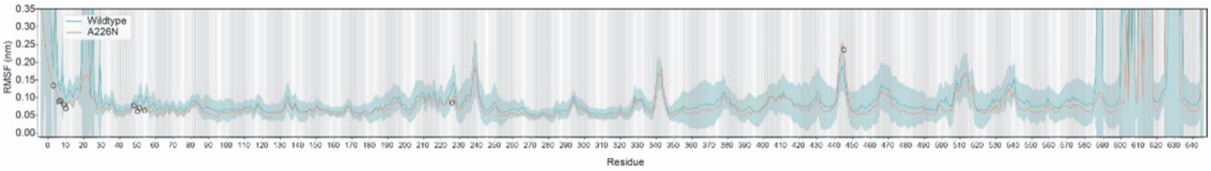

Figure S1. Root mean square fluctuations. Five independent MD simulations were executed for the wildtype and the A226N mutant, and the RMSF were calculated each simulation. The average RMSFs of the wildtype (in blue line) and A226N (in red line) were plotted with the 95% confidence interval (*t*-test) in shade. The residues with statistically significant changes were marked with empty circles. Generally, the mutation reduced RMSF in most residues but without statistical significance.

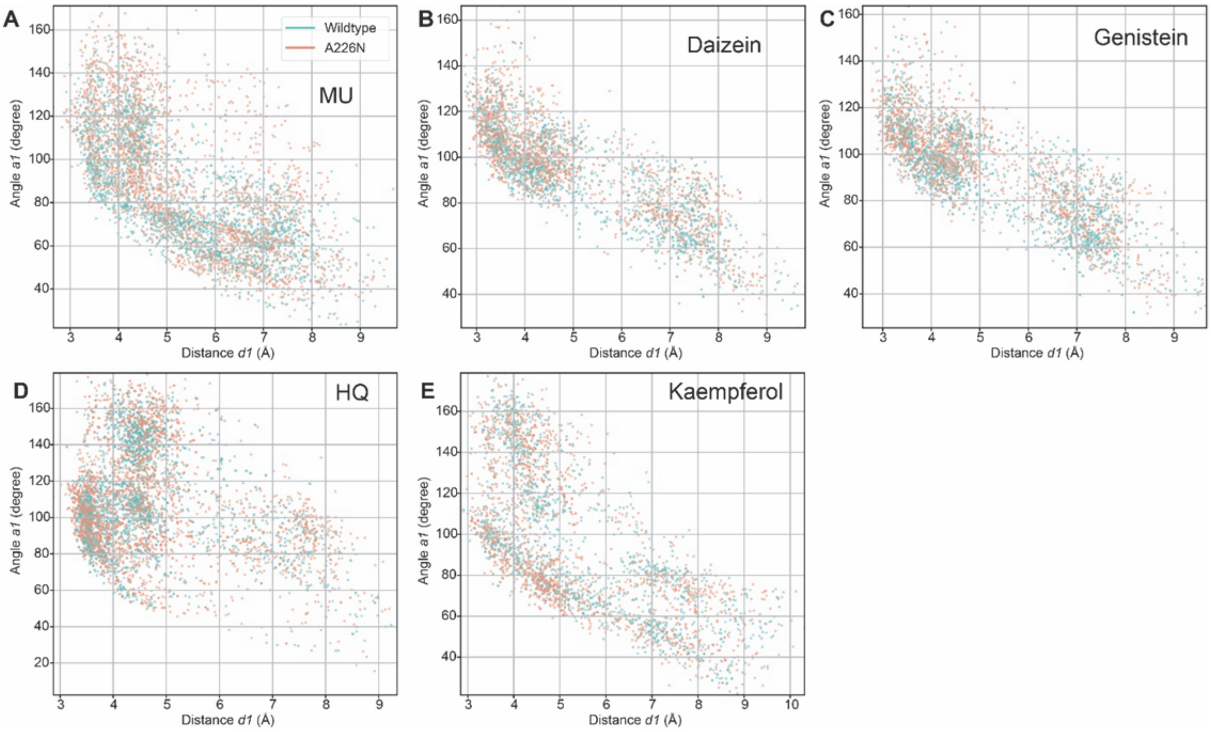

Figure S2. Docking conformations in *d1* and *a1* axes. For each docking conformation *d1*, *d2* and *a1* were calculated, and it was plotted on *d1* and *a1* axes. Only the conformations with d2 < 4Å were plotted. The docking conformations for the wildtype and the A226N mutant ensembles were plotted in blue and red spots, respectively. Each subplot represents the docking results for (A) MU, (B) daizein, (C) genistein, (D) HQ and (E) kaempferol.

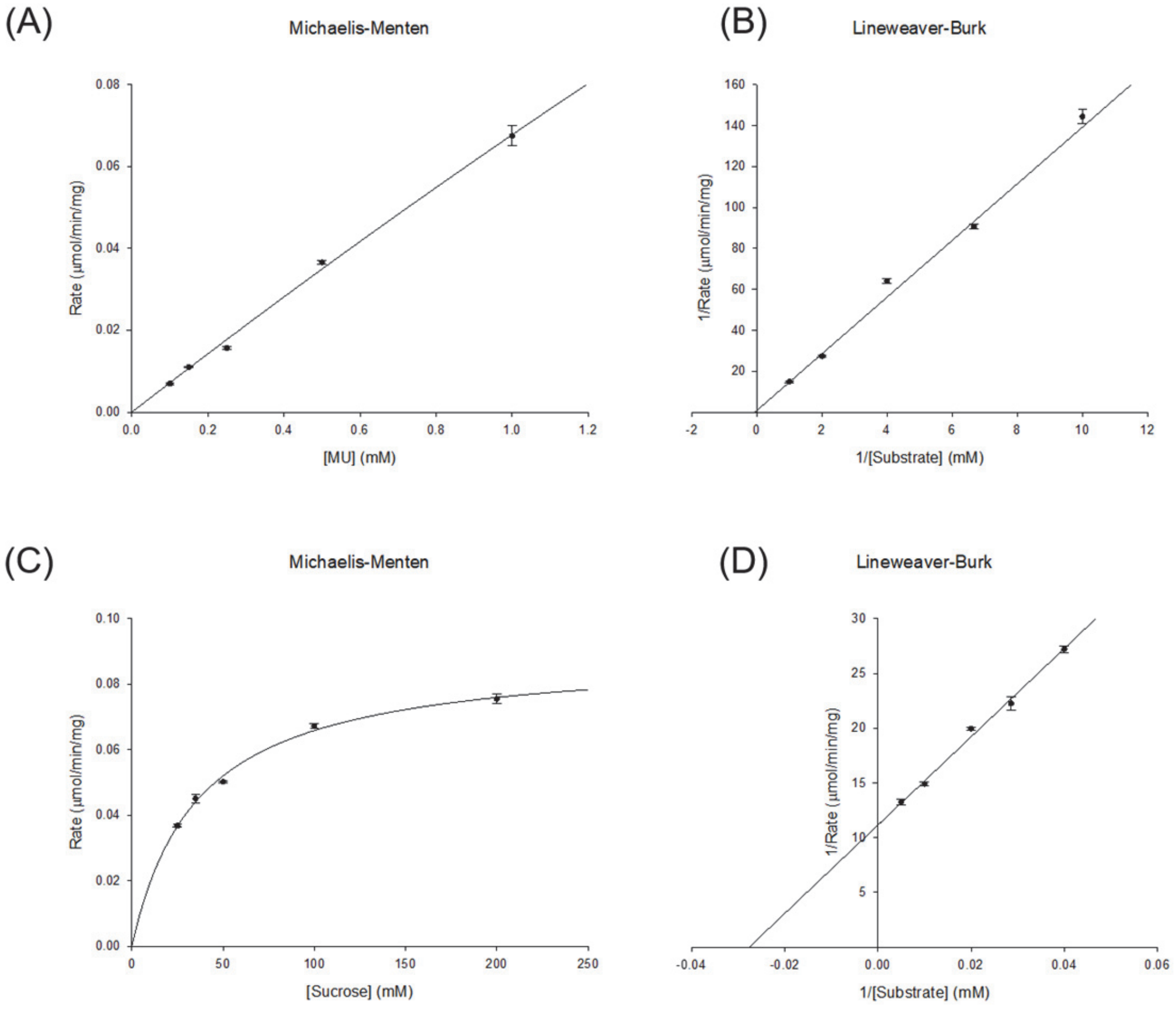

Figure S3. Michaelis-Menten (A,C) and Lineweaver-Burck (B,D) plots on the transglycosylation activity of DGAS with varied concentration MU (A, B) and varied concentration sucrose (C, D)

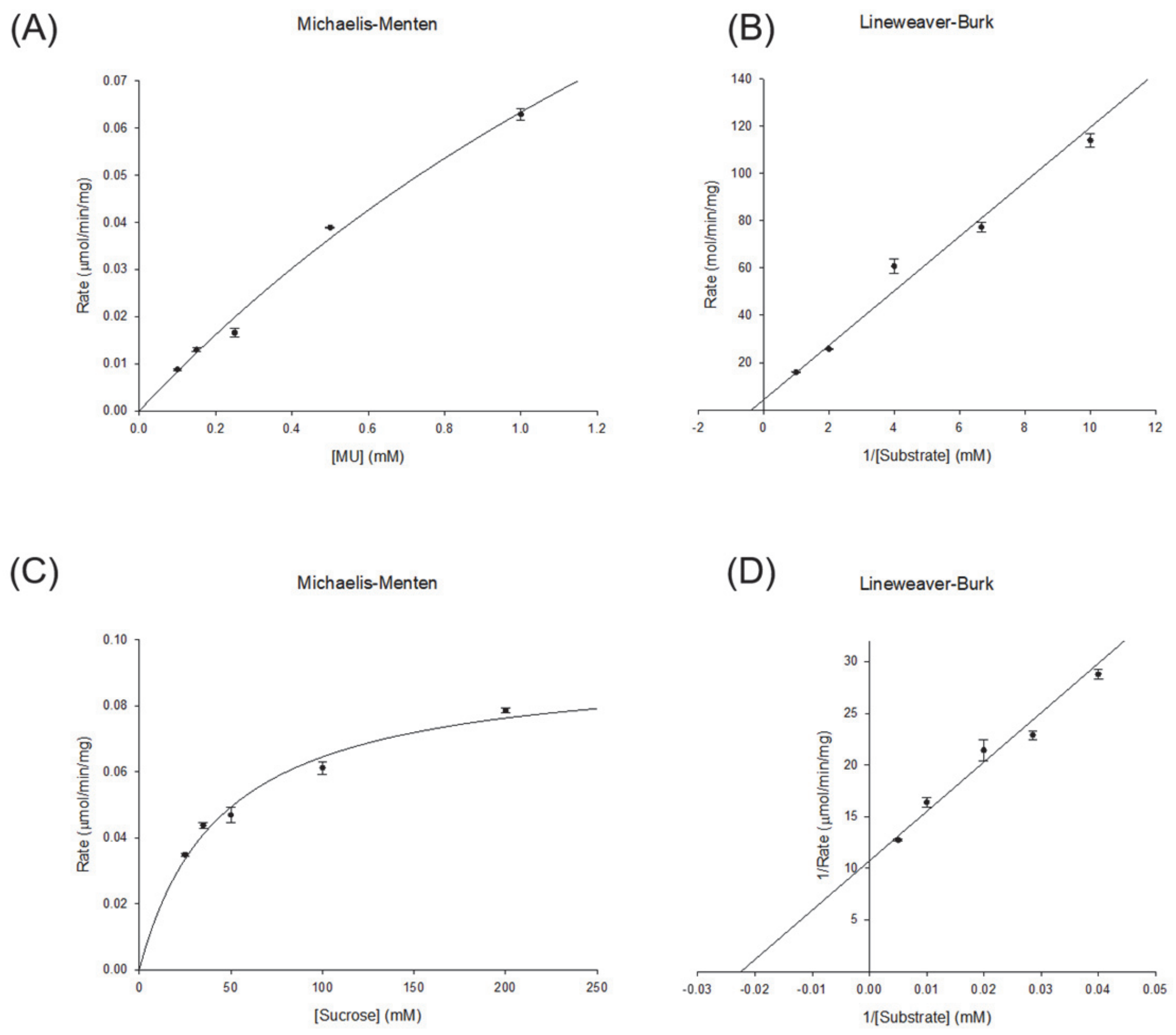

Figure S4. Michaelis-Menten (A,C) and Lineweaver-Burck (B,D) plots on the transglycosylation activity of DGAS-A226N with varied concentration MU (A, B) and varied concentration sucrose (C, D)
